# DynaTCR: dynamic hard-negative ensemble graph learning improves TCR-epitope binding prediction

**DOI:** 10.1093/bioinformatics/btag544

**Published:** 2026-07-21

**Authors:** Xiangzheng Fu, Xinyu Zhang, Linlin Zhuo, Yifan Chen, Dongsheng Cao, Quan Zou

**Affiliations:** Faculty of Computer Science and Artificial Intelligence, Shenzhen University of Advanced Technology, Shenzhen 518107, China; School of Data Science and Artificial Intelligence, Wenzhou University of Technology, Wenzhou 325000, China; School of Data Science and Artificial Intelligence, Wenzhou University of Technology, Wenzhou 325000, China; College of Computer and Mathematics, Central South University of Forestry and Technology, Changsha, Hunan 410004, China; School of Information Engineering, Changsha Medical University, Changsha 410219, China; Xiangya School of Pharmaceutical Sciences, Central South University, Changsha 410003, China; Institute of Fundamental and Frontier Sciences, University of Electronic Science and Technology of China, Chengdu 610054, China

## Abstract

**Motivation:**

T-cell receptors (TCRs) recognize antigenic peptides presented by major histocompatibility complex (MHC) molecules and are central to adaptive immunity. Computational prediction of TCR–epitope binding (TEB) can accelerate immunotherapy development, yet remains hampered by limited labeled data, false-negative noise in unobserved pairs, and over-smoothing in graph-based models.

**Results:**

We present DynaTCR, a dynamic graph ensemble learning framework for TEB prediction. DynaTCR encodes TCR and epitope sequences with protein language model embeddings and organizes them into a bipartite interaction graph. A graph regularization-variance-preserving aggregation (GR-VPA) encoder stabilizes message propagation and alleviates over-smoothing, while a global attention layer captures long-range dependencies. Multiple base learners are trained with iteratively updated hard-negative samples to reduce false-negative predictions. Under the StrictTCR evaluation protocol on four public datasets, DynaTCR achieves AUC improvements of 4.0–8.2 percentage points over the strongest existing method and up to 15.8 percentage points in AUPR. On the most stringently curated dataset, DynaTCR attains an AUC of 95.1%. Furthermore, on an independent structure-derived test set, DynaTCR achieves the highest AUC (72.6%) among all compared methods, demonstrating its robustness and effectiveness for TEB prediction and candidate prioritization.

**Availability:**

Source code and data can be downloaded from: https://github.com/2014402680/TEB/.

## 1 Introduction

T cells are central effectors of adaptive immunity. They recognize short peptide fragments-antigenic epitopes-presented on the cell surface by major histocompatibility complex (MHC) molecules, thereby initiating downstream signaling cascades that eliminate pathogens, virus-infected cells, and tumor cells (Rossjohn *et al*. 2015, Dens *et al*. 2023). In the context of tumor immunology, peptide neoantigens derived from somatic mutations constitute a major class of MHC-presented targets for T cell–mediated immune surveillance (Dunn *et al*. 2004). Accurate characterization of T cell–epitope binding (TEB) is therefore of broad significance, underpinning applications that range from neoantigen-based cancer vaccine design and adoptive T-cell therapy to autoimmune antigen identification (Chandran and Klebanoff 2019). Despite its importance, experimental determination of TEB specificity remains low-throughput and resource-intensive, as current assays such as peptide-MHC tetramer staining and T-cell functional assays involve high reagent costs and lengthy protocols. This bottleneck has motivated the development of computational approaches capable of predicting TEB at scale.

Computational models have been developed to simulate the interaction dynamics between T-cell receptors (TCRs) and antigenic epitopes. In adaptive immune responses, major histocompatibility complex (MHC) molecules present peptides on the cell surface for TCR recognition. The primary binding interface is predominantly between the complementary determining region 3 of the TCR β chain (CDR3β) and the antigenic epitope (Dens *et al*. 2023). High-throughput tetramer TCR sequencing technologies (Kula *et al*. 2019) and other experimental methods have accelerated the accumulation of TEB data, now cataloged in public databases such as VDJdb (Shugay *et al*. 2018), IEDB (Vita *et al*. 2019), and McPAS-TCR (Tickotsky *et al*. 2017). Despite these advances, current datasets remain limited compared to TCR diversity and exhibit imbalance due to epitope promiscuity, complicating computational predictions.

Machine learning-based approaches have significantly advanced TEB prediction (Fu *et al*. 2025). Early studies focused on constructing epitope-specific models to decipher TCR binding patterns for individual epitopes. These methods evolved from traditional sequence alignment to more sophisticated machine learning techniques, including random forests (Gielis *et al*. 2019) and Gaussian process classifiers (Jokinen *et al*. 2019). However, these approaches require developing separate models for each epitope and depend on large amounts of epitope-specific TCR data, which are often difficult to obtain. To address these limitations, researchers have developed universal models capable of predicting TCR binding to any peptide-MHC (pMHC) epitope. Recent deep learning frameworks have demonstrated notable improvements in this domain. TEINet (Jiang *et al*. 2023) employs transfer learning with pre-trained encoders for TCR and epitope sequences, enhancing prediction accuracy. TEIM (Peng *et al*. 2023) leverages large-scale sequence data for pre-training and captures spatial interactions among key residues during fine-tuning, thereby improving model generalization. pMTnet (Lu *et al*. 2021) uses a staged training strategy with feature embedding to address high-dimensional complexity, optimizing performance for data-scarce and imbalanced scenarios. Additionally, GTE (Jiang *et al*. 2024) integrates GNNs to model topological relationships between TCRs and epitopes, further improving prediction accuracy.

Deep learning has become pivotal for predicting TCR–epitope binding specificity, yet existing methods exhibit high false-negative rates in identifying novel binding events. To address this, we propose a dynamic graph ensemble learning model for TEB recognition. Our approach leverages the ESM-2 (Lin *et al*. 2022) model to extract sequence features from TCRs and epitopes, constructing high-quality TCR-epitope graphs. During training, multiple base models undergo dynamic negative sampling, with parameters co-optimized via ensemble learning, particularly prioritizing misclassified samples. This strategy reduces false negatives and enhances novel TEB discovery. We further introduce a novel GNN encoder combining variance-preserving aggregation (VPA) and graph regularization. This design preserves feature variance during message propagation, mitigating over-smoothing in sparse neighborhoods. A global attention mechanism is integrated to model long-range dependencies and optimize feature propagation. The main contributions of this work are summarized as follows:

- We propose DynaTCR, a unified graph-based framework for TEB prediction that formulates TCR-epitope interactions as a bipartite graph with ESM-2 derived sequence embeddings and introduces a novel graph encoder integrating graph regularization, variance-preserving aggregation, and global attention, which jointly stabilizes message propagation, alleviates over-smoothing, and captures long-range dependencies.
- We design a dynamic hard-negative ensemble learning strategy that iteratively re-weights difficult unobserved TCR-epitope pairs during training, effectively mitigating the impact of false-negative noise and improving discrimination under sparse supervision.
- Extensive experiments on four public TEB benchmarks under the StrictTCR evaluation protocol demonstrate that DynaTCR consistently outperforms competitive baselines across all metrics, confirming its effectiveness and robustness for both seen and unseen epitope settings.

## 2 Materials and methods

### 2.1 Data preparation

This study evaluated model performance using four public datasets: TEINet (Jiang *et al*. 2023), pMTnet (Lu *et al*. 2021), VDJdb (Shugay *et al*. 2018), and McPAS-TCR (Tickotsky *et al*. 2017). To ensure data reliability and consistency across different sources, rigorous filtering criteria were applied to the raw data (e.g. restricting CDR3 lengths to 5–30 amino acids, limiting epitope lengths to 7–15 amino acids, and retaining only high-confidence human MHC class I interactions).

The detailed data preprocessing pipelines and specific filtering thresholds for each dataset are comprehensively documented in Supplementary Section S1, available as supplementary data at *Bioinformatics* online. The final statistics of the curated datasets used for model training and evaluation are summarized in Table 1.

**Table 1:** Summary of the curated datasets used in this study.

Datasets	TCRs	Epitopes	TCR-Epitope Pairs
TEINet	41 610	180	44 682
pMTnet	28 604	426	31 203
VDJdb	36 033	1002	38 533
McPAS-TCR	4975	244	5102

### 2.2 Model architecture

**Figure 1:**
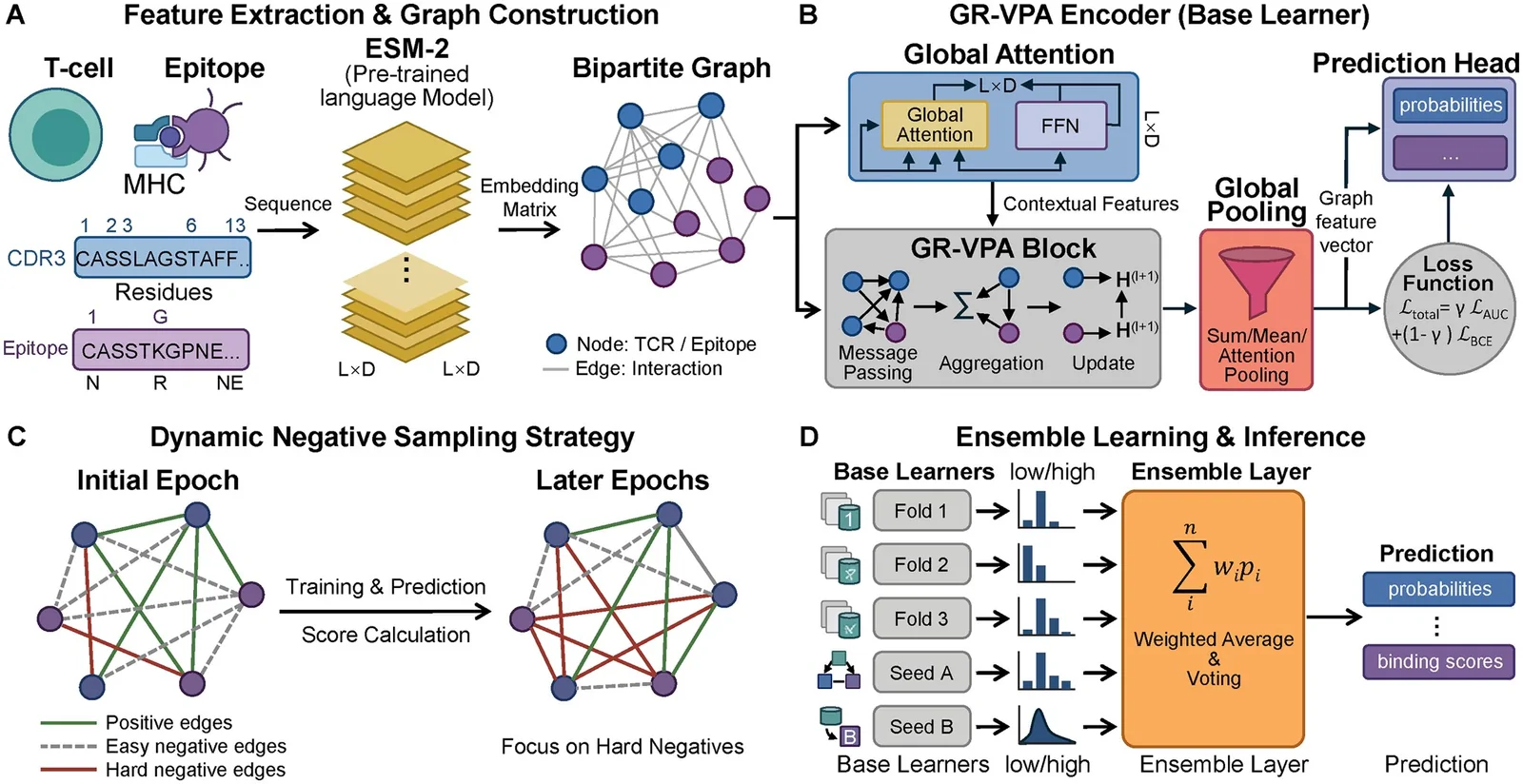
Overview of the DynaTCR framework. (A) Feature extraction and graph construction: CDR3β and epitope sequences are encoded by ESM-2 into sequence embeddings and organized into a TCR–epitope bipartite graph, in which TCRs and epitopes are heterogeneous nodes and observed interactions are edges. (B) GR-VPA encoder (base learner): a GR-VPA block performs message passing, variance-preserving aggregation, and graph regularization, followed by a global attention layer for long-range context modeling, with graph-level representations pooled for binding prediction. (C) Dynamic negative sampling strategy: prediction scores are iteratively used to shift training focus from easy to hard negative edges. (D) Ensemble learning and inference: multiple diversified base learners are aggregated via weighted averaging and voting to produce final binding predictions.

The architecture and operational workflow of the DynaTCR model are illustrated in Fig. 1. (A) TCR-epitope graph construction: The ESM-2 model utilizes TCR and epitope sequence embeddings to generate universal representations, followed by the construction of a bipartite graph from TCR-epitope paired data. (B) Graph regularization-VPA encoder: A base learner module integrating variance-preserving aggregation (VPA) and graph regularization for topological feature extraction. (C) DynaTCR workflow: The pipeline comprises: (i) TCR-epitope graph construction; (ii) topological feature extraction via the graph regularization-VPA encoder; (iii) multi-base model training with dynamic negative sampling and joint parameter optimization; and (iv) end-to-end prediction. (D) Execution example: A case study demonstrating the model’s application for TEB prediction, including input data processing and output interpretation.

### 2.3 Feature extraction based on ESM

The analysis of TCR and epitope sequences is challenging due to data scarcity and short sequence lengths. To obtain highly informative representations, we leveraged ESM-2 (Lin *et al*. 2022), a state-of-the-art protein language model, to extract high-dimensional feature vectors from both TCR and epitope sequences. Unlike frameworks optimized solely on specific TCR datasets, our architecture is tailored to maximize the utility of universal ESM-2 embeddings for predicting TCR-epitope interactions. Notably, our experiments indicate that utilizing appropriately sized ESM models prevents the introduction of noise, yielding optimal feature extraction without requiring massive computational overhead.

### 2.4 Constructing a TCR-epitope bipartite graph

In biological systems, protein-protein interaction (PPI) networks are critical for information transfer and functional collaboration (Maslov and Sneppen 2002, Gao *et al*. 2023). However, many existing methods focus on learning sample embeddings and distance metrics in feature space, neglecting the expressive power of network topology. This oversight may limit the model’s ability to characterize complex biological mechanisms.

In this study, the topological structure of the graph was introduced in TEB prediction. Unlike homogeneous PPI networks (Gao *et al*. 2023), TCRs and epitopes exhibit distinct biological properties and are modeled as heterogeneous nodes. As shown in Fig. 1, we construct a heterogeneous graph ***G*= (*N, E, X*)**, where ***N*** denotes the node set containing TCR and epitope nodes, and the edge set ***E*** represents edges defining relationships between these nodes. Edges are labeled 1 for positive interactions and 0 for negative interactions, with a single edge type used for consistency. This formulation frames the task as an edge classification problem. The feature matrix **X** contains node representations, where each node $v_{i}$ is associated with a feature vector $x_{i}$ extracted from a pre-trained ESM-2: $x_{i}$**=*ESM2*(**$n_{i}$**)**. In the constructed graph, edges $e_{\mathrm{ij}}$ connect nodes $n_{i}$ and $n_{j}$, with labels $y_{\mathrm{ij}}$ indicating binding interactions (1) or absence thereof 0. Formally, the edge set ***E*** and the label set ***Y*** are defined as follows:


(1) $$E=\{(n_{i},n_{j})\backslash n_{i}\in N,n_{j}\in N\},Y=\{y_{\mathrm{ij}}\backslash e_{\mathrm{ij}}\in E\}.$$

Through the above steps, we constructed a TCR-epitope bipartite graph to provide a more structured representation and lay the foundation for subsequent interaction prediction tasks.

### 2.5 Graph regularization-VPA encoder

GNNs have demonstrated considerable advantages in the analysis of network data, primarily due to their exceptional capacity to interpret topological structures. Nevertheless, conventional GNN architectures encounter several intrinsic limitations when applied to TEB prediction. As the number of layers in these networks increases, node embeddings often converge, resulting in diminished discriminative power and the prevalent issue of over-smoothing. Furthermore, the instability of gradients significantly hampers the efficiency of message propagation, while the predominance of high-degree nodes within the TCR-epitope graph exacerbates the imbalance in message propagation. Additionally, the traditional GNN’s reliance on local neighborhood aggregation reveals a notable inadequacy in capturing long-range interactions among TCR-epitopes, thereby severely restricting the model’s capacity to identify global interaction patterns.

To mitigate these challenges, we proposed a novel graph regularized-VPA encoder. This encoder addresses the dominance of high-density nodes during the message-passing process by implementing graph regularization constraints. Secondly, it incorporates VPA technology, which employs an adaptive aggregation strategy during message propagation to preserve the variance of node embeddings, thus preventing feature degradation and alleviating the over-smoothing phenomenon. Concurrently, the VPA mechanism ensures the stability of embedding variance throughout information propagation, thereby reducing the issues of gradient explosion or vanishing gradients that often arise in GNNs during deep propagation. Moreover, the integration of a global attention mechanism enables the model to effectively capture long-range dependency information across local neighborhoods, thereby enhancing its ability to interpret the overall topological information. In conclusion, the objective of this graph encoder is to optimize message propagation within the TCR-epitope graph, ensure the stability of the embedding space, and enhance the interpretation of information within the TCR-epitope graph.

### 2.6 Graph regularization and variance-preserving aggregation

In the TCR-epitope graph, epitopes typically exhibit higher neighborhood density than TCRs. To prevent high-degree nodes from dominating message propagation, we implement a graph regularization technique. This approach constrains the influence of dense nodes and ensures a balanced information flow, thereby optimizing the message propagation mechanism across the heterogeneous graph.

Furthermore, traditional GCNs often suffer from over-smoothing as network depth increases, causing node embeddings to converge. To address this, we incorporate a Variance-Preserving Aggregation (VPA) strategy (Schneckenreiter *et al*. 2024). VPA adaptively modifies the aggregation function to explicitly maintain the variance of node embeddings throughout the message-passing process. This mechanism not only prevents feature degradation but also stabilizes training by mitigating gradient explosion or vanishing issues during deep propagation. The complete mathematical formulations and derivations for both the graph regularization and the VPA mechanism are detailed in Supplementary Section S2, available as supplementary data at *Bioinformatics* online.

### 2.7 Global attention mechanism

In TEB prediction, modeling the complex biological relationships between heterogeneous sequences is challenging when relying solely on local topological information. To enhance the model’s ability to capture global dependencies, we introduce a global attention mechanism. This mechanism allows each node in the TCR-epitope bipartite graph to dynamically focus on information from all other nodes, effectively capturing long-range dependencies and optimizing cross-scale feature representations.

Specifically, we employ a standard scaled dot-product attention mechanism to update the initial feature vectors extracted by ESM-2. By computing attention scores across all node pairs, the model adaptively integrates global contextual semantic information. The detailed mathematical formulation of the query, key, and value projections, as well as the attention score matrix calculations, are provided in Supplementary Section S3, available as supplementary data at *Bioinformatics* online.

### 2.8 Ensemble learning with dynamic negative sampling

Treating all unobserved TCR-epitope pairs as negative samples introduces noise and weakens model discrimination. To address this, we integrated a dynamic negative sampling strategy within an ensemble learning framework.

Initially, the training set is constructed with an equal proportion of positive and negative samples. During training, positive samples remain fixed, while negative samples are dynamically updated: misclassified negative samples are retained to strengthen the model’s ability to recognize difficult cases, and correctly predicted negative samples are resampled at a specific proportion (ϵ) to introduce progressively challenging data. At each update step, negative samples predicted as positive by the current model are retained as hard negatives, whereas a proportion ε of correctly classified negatives is replaced by newly sampled negatives from the unknown TCR-epitope pool. Here, ε denotes the replacement ratio of correctly classified negatives in each update round. This module operates during training rather than post-processing: it iteratively updates the negative sample distribution and thereby refines the optimization of the backbone predictor.

To enhance diversity within the ensemble, m base learners are trained on iteratively updated negative sets generated during different training rounds. Rather than directly optimizing one another’s parameters, these base learners are independently trained under different negative-sample configurations, allowing the ensemble to capture complementary decision boundaries. During inference, the predictions of these base learners are aggregated using a weighted strategy optimized on the validation set, which enables the final model to integrate complementary information learned from diverse negative-sample distributions.

### 2.9 Deep AUC maximization

Limited TEB data restricts model learning and generalization. While introducing more negative samples expands the data scale, it risks causing severe data imbalance, a challenge that our dynamic negative sampling strategy may further exacerbate (Jurtz *et al*. 2018). To evaluate and optimize model performance effectively under such imbalanced conditions, we adopt Deep AUC Maximization (Yuan *et al*. 2021).

Unlike standard loss functions that optimize overall accuracy, Deep AUC Maximization directly optimizes the Area Under the ROC Curve (AUC), which measures the model’s ability to rank positive samples strictly above negative ones. By formulating the AUC optimization as a stochastic saddle point problem, the model maintains robust discriminative power regardless of the positive-to-negative sample ratio. The detailed mathematical derivation of the AUC loss, denoted as $L_{\mathrm{AUC}}$, is provided in Supplementary Section S4, available as supplementary data at *Bioinformatics* online.

To comprehensively train the DynaTCR model, the final objective function combines standard Binary Cross-Entropy (BCE) loss with the AUC loss:


(2) $$L=\gamma \times L_{\mathrm{AUC}}+(1-\gamma )\times L_{\mathrm{BCE}},$$

Where γ is a hyperparameter that controls the weighting of different components to balance the trade-off. BCE loss mainly improves sample-wise classification calibration and probability fitting, whereas AUC loss emphasizes the pairwise ranking relationship between positive and negative samples. Their combination allows the model to balance local classification quality and global ranking performance, which is particularly beneficial under noisy and class-imbalanced settings.

## 3 Results

This study introduces the comparative model and its experimental configurations. The performance of the DynaTCR and the comparative models is evaluated on four public datasets. Ablation and parameter experiments are conducted to assess the contribution of each key module and the model’s sensitivity to parameter changes. The model’s generalization ability is further validated through additional evaluations on an independent test set. Case studies are also performed to analyze the process of detecting key contact sites from the TCR pool. Parameter sensitivity analysis (Supplementary Section 5 and Fig. S1, available as supplementary data at *Bioinformatics* online) demonstrates that DynaTCR is highly robust to variations in AUC loss weight **(**γ**)** and dynamic sampling probability **(**ε**).** 

### 3.1 Experimental setup

In TEB specificity prediction, five-fold cross-validation is commonly used to evaluate model performance (Lu *et al*. 2021, Jiang *et al*. 2023, Peng *et al*. 2023). Traditional methods often use random data splitting, which may cause data leakage, such as repeated TCR sequences appearing in both the training and test sets. This can lead to unreliable evaluation results and overestimated model performance. Therefore, this study adopts the StrictTCR strategy proposed by Jiang *et al*. (2024). This method ensures that the same TCR does not appear in both the training and test sets by grouping data based on unique TCR identifiers. Additionally, it avoids overlap of negative samples between the training and validation sets during negative sample generation.

In our implementation, the graph encoder consists of three layers with a hidden dimension of 3, and the global attention module uses three attention heads. The model is optimized using Adam with a learning rate of 0.001. In addition, the ensemble framework includes five base learners. All hyperparameters were selected through five-fold cross-validation on the training set.

### 3.2 Performance comparison

We systematically evaluated the performance of the DynaTCR against various mainstream baseline models on four datasets: TEINet (Jiang *et al*. 2023), VDJdb (Shugay *et al*. 2018), pMTNet (Lu *et al*. 2021), and McPAS (Tickotsky *et al*. 2017). Table 2 presents the results of five-fold cross-validation under the StrictTCR split strategy. Our model demonstrates superior performance compared to baseline methods, particularly on TEINet and VDJdb. Specifically, it achieves an 8 percentage point improvement in AUC on TEINet and a 15 percentage point improvement in AUPR on VDJdb, both significantly surpassing existing baselines. Notably, while the GTE model also leverages topological information, it uses the TCRpeg method for embedding generation. TCRpeg is a pre-trained model based on dataset training, containing 106 TCR sequences and 362 456 unique epitopes. However, its generalization ability is limited by the diversity and coverage of its training data. In contrast, our method employs ESM-2, a large-scale protein language model, which significantly enhances embedding generalization and expressiveness. The experimental results strongly validate our approach, which combines high-quality embeddings from a universal pre-trained model with a novel global attention-based topological architecture, demonstrating the architecture’s effectiveness and advantages.

**Table 2:** The performance of DynaTCR and the baseline model across four datasets (%).

Datasets	TEINet		pMTNet		VDJdb		McPAS	
methods	AUC	AUPR	AUC	AUPR	AUC	AUPR	AUC	AUPR
TEIM	63.5	21.1	63.7	17.5	61.7	20.2	69	31.6
TEINet	60.7	12.9	60.8	13.1	58.1	11.8	66.2	23.7
PMTnet	61.6	15.3	63.4	16.5	60.2	16.0	64.5	19.3
GTE	83.9	48.6	91.1	65.5	84.7	50.0	81.3	39.0
DynaTCR	92.1	63.5	95.1	74.2	92.8	65.8	88.2	49.0

On the pMTnet dataset, the DynaTCR method achieved an AUC score of 0.951 and an AUPR score of 0.742, significantly outperforming all baseline methods. This advantage may stem from the method’s ability to construct a high-quality TCR-epitope bipartite graph, enhancing its capacity for structural information modelling. Additionally, the DynaTCR method’s superior performance on pMTnet compared to other datasets is likely due to the dataset’s characteristics. pMTnet integrates data from multiple sources and retains only high-confidence samples (Glanville *et al*. 2017, Tickotsky *et al*. 2017, Shugay *et al*. 2018, Jokinen *et al*. 2021), while other datasets may include low-confidence samples that can negatively impact model performance.

Despite potential noise in the other three datasets, the DynaTCR still significantly outperformed all baseline models. This indicates that baseline methods may struggle to adapt to diverse data sources, particularly when handling low-confidence data. In contrast, the DynaTCR enhances robustness in low-quality data environments through graph regularization-VPA encoder optimization, dynamic negative sampling, and ensemble learning. These strategies improve the model’s adaptability and resilience, making it more effective in challenging data conditions.

Comparative analysis (Supplementary Section 6, Fig. S2, available as supplementary data at *Bioinformatics* online) confirmed that the ESM2-8M model provides the optimal balance of feature representation and computational efficiency compared to larger models or domain-specific TCRpeg.

### 3.3 Ablation experiment

The ablation study results, presented in Table 3, demonstrate the impact of removing key components from the model on four datasets. The table uses abbreviations such as “w/o GA” to indicate the removal of the global attention mechanism, “w/o DNS” denotes removing dynamic negative sampling, while“w/o NS”denotes removing dynamic negative sampling and using random sampling instead. “w/o ED” for the removal of ensemble learning based on dynamic negative sampling, “w/o AUC” for the removal of AUC loss, and “w/o GVN” for the removal of the graph regularization-VPA encoder. To further dissect the GR-VPA encoder, we additionally evaluated two separate variants, namely “w/o GR” and “w/o VPA”, in order to assess their individual contributions. The complete model is referred to as “DynaTCR.”

**Table 3:** Results of ablation experiments across four datasets (%).

Datasets	TEINET		pMTNet		VDJdb		McPAS	
Models/metrics	AUC	AUPR	AUC	AUPR	AUC	AUPR	AUC	AUPR
w/o GA	91.7	63	94.3	73.1	92.4	65.2	87.3	47.6
w/o AUC loss	91.6	62.9	94.6	73.6	92.2	65.3	87.8	48.0
w/o GR + VPA	91.6	62.7	94.1	72.7	92.0	65.1	87.5	47.6
w/o GR	91.8	62.9	94.8	73.6	92.4	65.3	87.8	48.3
w/o VPA	91.9	63.0	94.5	73.4	92.5	65.4	87.8	48.4
w/o ED	91.4	61.8	93.5	71.2	91.5	63.6	87.1	45.8
w/o NS	90.3	61.7	93.2	71.1	90.8	62.9	86.8	45.1
w/o DNS	90.8	61.2	93.4	71.2	91.0	63.4	86.9	45.5
DynaTCR	92.1	63.5	95.1	74.2	92.8	65.8	88.2	49.0

The results indicate that removing any key component leads to a decline in model performance, underscoring their importance in TEB prediction. For instance, on the pMTnet dataset, removing the global attention mechanism caused the AUC to drop from 95.1% to 94.3% and the AUPR to decrease from 74.2% to 73.1%, indicating that it makes a stable contribution to capturing TCR-epitope interaction patterns. Similarly, removing the graph regularization-VPA encoder or AUC loss resulted in performance declines across TEINET, VDJdb, and McPAS datasets, demonstrating their positive contributions to model performance. The most significant performance reduction occurred when dynamic negative sampling-based ensemble learning was removed, indicating it is the most impactful component. This finding further supports the strategy’s effectiveness in identifying true TEBs from unknown TCR-epitope pairs and reducing false negatives.

### 3.4 Evaluation on the independent dataset

This study utilized the independent test set PDB provided by the baseline model TEINet, which was derived from the RCSB Protein Database (PDB) (Burley *et al*. 2023). Following stringent screening of 105 TCR-epitope complex structures, 63 positive samples were retained for evaluation. The test set features a balanced data distribution, with each epitope binding to only one or two TCRs, which increases task difficulty and enables effective assessment of model adaptability in complex scenarios. Importantly, this PDB-derived benchmark provides a more stringent evaluation than conventional cross-validation because it is based on experimentally resolved TCR-epitope complex structures and exhibits lower epitope redundancy and weaker overlap with the training distribution. Therefore, performance on this dataset more directly reflects the model’s ability to generalize to biologically distinct and previously unseen interactions. To ensure experimental fairness, the TEINet dataset served as the training set, and negative samples were generated at a 1:1 ratio using the StrictTCR strategy, consistent with the approach applied to the PDB independent test set. All models underwent five-fold cross-validation to identify optimal hyperparameters, which were subsequently applied to the independent test set. This protocol was adopted because unobserved TCR-epitope pairs cannot be assumed to be true non-binders, and therefore computationally generated negative samples inevitably contain label noise. By using the StrictTCR strategy, we aimed to reduce over-optimistic evaluation caused by repeated TCR patterns and to provide a more rigorous assessment under uncertain negative labels.

As depicted in Fig. S3(b), available as supplementary data at *Bioinformatics* online, the DynaTCR outperformed all baseline models across all evaluation metrics, demonstrating exceptional generalization ability and stability. This outcome indicates that the DynaTCR effectively captures intricate interaction patterns between TCR and epitope sequences, enabling robust predictive performance on unseen data. Moreover, the model’s ability to maintain high generalization accuracy in an isolated test environment further validates the rationality of its structural design and its practical utility. Collectively, these results confirm that the DynaTCR can accurately analyze TEB specificity, providing strong methodological support for immune repertoire analysis tasks.

### 3.5 Case studies

#### 3.5.1 Detection of key contact sites from TCR pools

Beyond binary binding prediction, DynaTCR can effectively infer the binding conformation and reveal key contact points between TCRs and epitopes. To verify this capability, we compared the contact scores predicted by DynaTCR with the enriched CDR3 β motifs identified by the GLIPH method (Glanville *et al*. 2017). As illustrated in Fig. S3, available as supplementary data at *Bioinformatics* online, the predicted contact scores for GLIPH-identified motif regions were significantly higher than those for non-motif regions. This consistency demonstrates that DynaTCR accurately captures the critical contact sites governing epitope binding, validating the structural importance of these motifs in TCR-epitope interactions (detailed case analyses are provided in Supplementary Section S7, available as supplementary data at *Bioinformatics* online).

#### 3.5.2 Application in virtual screening for retinitis pigmentosa-associated antigens

To further validate the applicability of DynaTCR in a realistic biological context, we conducted a virtual screening experiment focusing on Retinitis Pigmentosa (RP), a degenerative eye disease. We selected Rhodopsin (RHO, UniProt ID: P08100), a primary autoantigen associated with RP, as the target protein. Specifically, we extracted an immunogenic epitope fragment, AAYMFLLIV, from the cytoplasmic tail of RHO, which has been previously implicated in G protein-coupled receptor signaling and immunogenicity (Zhou *et al*. 2017).

We constructed a candidate TCR pool consisting of 20 human TCR β-chain CDR3 sequences retrieved from the VDJdb database. These sequences were originally annotated as binders to diverse antigens, including Influenza, COVID-19, and various autoantigens, representing a heterogeneous immune repertoire. The pre-trained DynaTCR model was employed to predict the binding probability between these TCRs and the RHO epitope.

##### 3.5.2.1 Evaluation on RHO epitope

Supplementary Table S1, available as supplementary data at *Bioinformatics* online presents the comparative analysis of DynaTCR predictions on Retinitis Pigmentosa-associated antigens. The prediction results indicated a high binding potential for the RHO epitope across the candidate pool. The binding scores ranged from 0.647 to 0.905, with a mean score of 0.822. Notably, four TCR sequences achieved scores exceeding 0.85, suggesting strong potential interaction. The highest-scoring sequence was CASTGTGVFNEQFF (0.905), followed by CASSEGGGEQYF (0.866) and CASGGNSYEQYF (0.861). These findings not only corroborate the high immunogenic potential of the selected RHO epitope but also demonstrate the effectiveness of DynaTCR in prioritizing high-affinity TCR candidates from a mixed repertoire.

##### 3.5.2.2 Evaluation on PDE6B epitope (specificity check)

To assess the model’s specificity and generalization ability across different retinal antigens, we extended the experiment to Phosphodiesterase 6B (PDE6B, UniProt ID: P16499), another key target in retinal degeneration. We tested the same pool of 20 TCR sequences against the PDE6B epitope VEDVAECPHF.

In contrast to the RHO experiment, the predicted scores for the PDE6B epitope were markedly lower, clustering between 0.579 and 0.776, with a mean score of 0.718. Only one sequence (CSADPVAGGGYEQYF) exceeded a threshold of 0.75. The significant difference in score distributions between RHO and PDE6B suggests that DynaTCR can effectively discriminate between epitopes with varying immunogenic profiles or binding affinities.

Collectively, these comparative experiments highlight two critical capabilities of DynaTCR: (1) the ability to identify potential high-affinity binders for specific autoantigens, and (2) the capacity to maintain selectivity, avoiding over-generalization across different targets. This positions DynaTCR as a promising computational tool for investigating potential cross-reactivity and screening immunogenic targets in autoimmune diseases.

## 4 Discussion

Early TCR-epitope prediction models were heavily dependent on large-scale paired training data; however, the scarcity of high-quality samples significantly restricted their applicability. Recent frameworks such as pMTnet and TEINet have demonstrated promise in immunotherapy prediction, yet they confront two critical challenges: (1) traditional protein modelling approaches (e.g. multiple sequence alignment, MSA) exhibit limitations in capturing short-sequence interactions; (2) single-sequence language models (e.g. ESM-2) suffer from constrained transferability when applied to TE tasks. Although GNNs have advanced the field, they remain hindered by data sparsity and the over-smoothing problem in message propagation. To address these gaps, we propose a TEB prediction model integrating protein language models and ensemble GNNs. Specifically, we develop an ESM2-based TCR-epitope graph construction method, which extracts contextual representations of TCR and epitope sequences to build interaction graphs. A synergistic optimization mechanism is designed using dynamic negative sampling and ensemble learning, focusing on misclassified hard samples to reduce false-negative predictions. Because unknown TCR-epitope pairs are not guaranteed to be true negatives, this strategy is intended to mitigate the impact of noisy negative labels rather than treating all unobserved pairs as equally reliable non-binders. By iteratively emphasizing more informative hard negatives, the model can better reduce the interference of potential false negatives during training. Additionally, a GNN encoder incorporating variance-preserving aggregation and graph regularization is introduced to optimize message propagation, effectively mitigating the over-smoothing issue. Experimental results on four benchmark datasets demonstrate substantial performance gains: our model achieves an 8% increase in AUC and a 15% increase in AUPR on the TEINet dataset. Ablation studies further validate the critical contributions of each proposed component. However, the larger performance drop observed on the independent PDB test set suggests that the proposed graph-based framework may be more sensitive to distribution shift, particularly when the test data contain structurally distinct and less redundant TCR-epitope patterns than those observed during training.

Like current state-of-the-art methods, the DynaTCR model primarily leverages CDR3 region information from the TCR β chain for modelling. However, evidence suggests that exclusive reliance on CDR3β fragments may impede predictive performance, as this region’s limited sequence diversity cannot fully capture the complex features of TCR-epitope interactions. In contrast, integrating paired information from both TCR α and β chains can provide more comprehensive feature representations and enhance prediction accuracy. Several additional limitations should also be noted. First, currently available public datasets may still contain epitope-frequency bias and residual redundancy, which could affect model evaluation and lead to overly optimistic estimates on frequently observed patterns. Second, the present study mainly focuses on human MHC-I-associated TCR β-chain CDR3 sequences, and the transferability of the model to other species, other MHC contexts, or full-length TCR settings remains to be further validated. Third, because unknown TCR-epitope pairs cannot be assumed to be true non-binders, label incompleteness remains an intrinsic challenge in TEB prediction and may introduce potential false-negative noise into both training and evaluation. Therefore, future research will explore modelling strategies for full-chain TCR sequences, aiming to design heterogeneous GNNs that fully exploit the complementary information of α/β chain interactions. Additionally, due to the computational complexity arising from ensemble learning and the Transformer architecture, future work will focus on optimizing efficiency by incorporating advanced protein pre-training models, along with model pruning, knowledge distillation, and quantization techniques. These efforts aim to improve the model’s scalability for large-scale datasets while maintaining its capability to capture intricate interaction patterns.

## 5 Conclusion

This study introduces an ensemble GNN framework for TEB prediction, integrating protein language models, dynamic negative sampling-based ensemble learning, and graph regularization with VPA to address challenges in sequence interaction modelling and generalization. The proposed model advances the field through three core innovations: leveraging the ESM-2 protein language model to extract contextual representations for constructing bipartite interaction graphs that capture latent sequence dependencies; employing dynamic negative sampling combined with ensemble learning to iteratively optimize base model parameters and reduce false-negative predictions, achieving up to a 15% improvement in AUPR on benchmark datasets; and designing a graph regularized-VPA encoder to maintain feature variance during message propagation, mitigating over-smoothing and yielding an 8% AUC improvement on the TEINet dataset, while a global attention mechanism enhances modelling of long-range interactions.

Extensive evaluations across four public datasets demonstrate that the model outperforms state-of-the-art baselines such as GTE and TEINet in both AUC and AUPR metrics, with robust generalization evident from an AUC of 72.6% on the independent PDB test set. Ablation studies confirm that dynamic negative sampling and ensemble learning are critical drivers of performance gains, underscoring the value of adaptive sample selection and collaborative model optimization. While this work represents a significant advancement, two limitations merit attention: the current model’s reliance on TCR β-chain CDR3 region information, which may restrict capture of complex binding conformations by neglecting α-chain synergies, and the computational complexity arising from ensemble learning and Transformer-based language model integration. In addition, the negative samples used in this study were computationally generated from unobserved TCR-epitope pairs and may still contain latent positives. Although dynamic hard-negative ensemble learning helps mitigate this issue, it cannot fully replace experimentally validated non-binders. Future work will incorporate higher-confidence non-binding data when such resources become available. Future research will focus on developing heterogeneous graph models to incorporate full-chain TCR sequence information and adopting lightweight techniques like knowledge distillation and model pruning to enhance scalability. These efforts aim to deliver more efficient and accurate tools for immunotherapy design, vaccine development, and autoimmune disease research, positioning the framework as a foundational step toward comprehensive TCR-epitope interaction modelling.

## Author contributions

Xiangzheng Fu (Conceptualization [Lead], Data curation [Equal], Funding acquisition [Equal], Investigation [Equal], Project administration [Equal], Writing—original draft [Equal], Writing—review & editing [Equal]), Xinyu Zhang (Data curation [Supporting], Software [Equal]), Linlin Zhuo (Formal analysis [Equal], Resources [Equal]), Yifan Chen (Investigation [Equal], Methodology [Equal], Project administration [Equal], Supervision [Lead], Validation [Equal]), Dongsheng Cao (Conceptualization [Equal], Methodology [Equal], Validation [Equal]), and Quan Zou (Project administration [Equal])

## Supplementary Material

btag544_Supplementary_Data
