# Supplementary material for: DynaTCR: dynamic hard-negative ensemble graph learning improves TCR-epitope binding prediction
Source: Bioinformatics. 2026 Jul 21;42(8):btag544. doi: 10.1093/bioinformatics/btag544 (PMC13450428; doi:10.1093/bioinformatics/btag544)
Supplement: Supplementary file 1 [file btag544_supplementary_data.docx]

**Supplementary Section S1: Detailed Data Preprocessing and Filtering Criteria**

The pMTnet dataset underwent rigorous quality control, retaining only high-confidence TCR-epitope pairs. The data primarily originate from various databases, including VDJdb and McPAS-TCR, and were filtered by vdj.score > 0 to enhance data reliability.

For the VDJdb dataset, which contains 89,847 filtered CDR3 sequences derived from TCR α and β chains of humans, monkeys, and mice, we applied rigorous filtering criteria to maintain consistency: we retained only entries associated with MHC class I, focused on β chain sequences, restricted CDR3 length to 5-30 amino acids, limited epitope length to 7-15 amino acids, and used only human data. The McPAS-TCR dataset is a manually curated collection of 40,033 TCR-epitope pairs from humans and mice. To ensure consistency with the VDJdb dataset, we applied the identical filtering criteria mentioned above.

**Supplementary Section S2: Mathematical Formulations of Graph Regularization and VPA**

**Graph Regularization Technique**

In the TCR-epitope graph, epitopes exhibit higher neighborhood density than TCRs, which can lead to high-degree nodes dominating message propagation and obscuring information from low-degree nodes. Drawing from prior research (Cho, 2024), this study implements graph regularization techniques to regulate both high-degree and low-degree nodes. This approach aims to enhance the efficiency of message propagation by ensuring a more balanced information flow within the graph structure.

Given a feature matrix $\boldsymbol{X=}\left[ \vec{\boldsymbol{x}_{\boldsymbol{1}}}\boldsymbol{,}\vec{\boldsymbol{x}_{\boldsymbol{2}}}\boldsymbol{,}\boldsymbol{\cdots,}\vec{\boldsymbol{x}_{\boldsymbol{n}}} \right]^{\boldsymbol{T}}$, where $\boldsymbol{x}_{\boldsymbol{i}}\boldsymbol{\in}\boldsymbol{R}^{\boldsymbol{m}}$ represents the content feature vector of node $\boldsymbol{v}_{\boldsymbol{i}}$ , and $\boldsymbol{n}$ is the total number of nodes. First, calculate the feature transformation vector based on the linear layer:

$\vec{\boldsymbol{h}_{\boldsymbol{i}}}\boldsymbol{=}\vec{\boldsymbol{x}_{\boldsymbol{i}}}\boldsymbol{Q}$, (1)

where $\boldsymbol{Q\in}\boldsymbol{R}^{\boldsymbol{m}\boldsymbol{\times f}}$ represent a learnable weight matrix. Let $\boldsymbol{c\in}\boldsymbol{R}$ be a scaling constant that constrains the implicit embedding space during the propagation process, generating normalized vectors:

$\vec{\boldsymbol{n}_{\boldsymbol{i}}}\boldsymbol{=c}\frac{\vec{\boldsymbol{h}_{\boldsymbol{i}}}}{\left\| \vec{\boldsymbol{h}_{\boldsymbol{i}}} \right\|}$, (2)

Subsequently, aggregate the normalized vectors of the neighborhood and itself to update its own embedding:

$\vec{\boldsymbol{z}_{\boldsymbol{i}}}\boldsymbol{=}\frac{\boldsymbol{1}}{\boldsymbol{d}_{\boldsymbol{i}}\boldsymbol{+1}}\vec{\boldsymbol{n}_{\boldsymbol{i}}}\boldsymbol{+}\sum_{\boldsymbol{j\in}\boldsymbol{N}_{\left( \boldsymbol{i} \right)}} \frac{\boldsymbol{1}}{\sqrt{\boldsymbol{d}_{\boldsymbol{i}}\boldsymbol{+1}}\sqrt{\boldsymbol{d}_{\boldsymbol{j}}\boldsymbol{+1}}}\vec{\boldsymbol{n}_{\boldsymbol{j}}}$, (3)

For the feature matrix $\boldsymbol{X\in}\boldsymbol{R}^{\boldsymbol{m}\boldsymbol{\times f}}$ and the adjacency matrix $\boldsymbol{K}$, the $\boldsymbol{GNCN(X,K,c)}$ is defined as follows:

$\boldsymbol{GNCN(X,K,c)=c}{\hat{\boldsymbol{D}}}^{\boldsymbol{-}\frac{\boldsymbol{1}}{\boldsymbol{2}}}\hat{\boldsymbol{K}}{\hat{\boldsymbol{D}}}^{\boldsymbol{-}\frac{\boldsymbol{1}}{\boldsymbol{2}}}\boldsymbol{g(XQ)}$, (4)

where$\boldsymbol{g}\left( \left[ \vec{\boldsymbol{h}_{\boldsymbol{1}}}\boldsymbol{,}\vec{\boldsymbol{h}_{\boldsymbol{2}}}\boldsymbol{,}\boldsymbol{\cdots,}\vec{\boldsymbol{h}_{\boldsymbol{n}}} \right]^{\boldsymbol{T}} \right)\boldsymbol{=}\left[ \frac{\vec{\boldsymbol{h}_{\boldsymbol{1}}}}{\left\| \vec{\boldsymbol{h}_{\boldsymbol{1}}} \right\|}\boldsymbol{,}\frac{\vec{\boldsymbol{h}_{\boldsymbol{2}}}}{\left\| \vec{\boldsymbol{h}_{\boldsymbol{2}}} \right\|}\boldsymbol{,\cdots,}\frac{\vec{\boldsymbol{h}_{\boldsymbol{n}}}}{\left\| \vec{\boldsymbol{h}_{\boldsymbol{n}}} \right\|} \right]^{\boldsymbol{T}}$, represents the node embedding matrix, and $\hat{\mathbf{D}}$ is the degree matrix of $\hat{\boldsymbol{K}}$. The integration of structural and content information is achieved by utilizing the graph's adjacency and feature matrices. To address the dominance of high-degree nodes, graph regularization technology is introduced, effectively constraining their influence and optimizing the message propagation mechanism. This approach ensures a more balanced information flow, enhancing the model's ability to analyze complex graph interactions.

**Variance-Preserving Aggregation (VPA)**

Previous research has indicated that an increase in the number of layers within GNNs can result in over-smoothing during the message passing phase, leading to the convergence of representations across different nodes and consequently diminishing the model’s discriminative capacity (Zhao and Akoglu, 2019). This phenomenon is also observed in the TCR-epitope bipartite graph, where conventional GCNs may cause the embeddings of TCRs or epitopes to become overly similar during propagation, thereby impairing the model's ability to differentiate between them.

To address this challenge, the VPA strategy (Schneckenreiter, et al., 2024) is employed, which adaptively modifies the aggregation approach to explicitly maintain the variance of node embeddings throughout the message passing process, thereby mitigating the over-smoothing issue. Additionally, the normalization aggregation mechanism inherent in VPA effectively reduces extreme fluctuations in activation values, thereby preventing problems related to gradient explosion or vanishing, and ensuring the stability of the model's training during deep propagation.

The fundamental concept of VPA involves the introduction of a novel aggregation function designed to preserve variance $\boldsymbol{m}_{\boldsymbol{i}}\boldsymbol{\sim N}\left( \boldsymbol{0,I} \right)$, which is accomplished through the aggregation function: $\frac{\boldsymbol{1}}{\sqrt{\boldsymbol{N}}}\sum_{\boldsymbol{i=1}} \boldsymbol{N}$. The traditional mean aggregation utilized in GCNs is enhanced to form the VPA aggregation, thereby preventing the loss of information or the collapse of distribution during feature propagation. Specifically, this can be formally expressed as follows:

$\boldsymbol{h}_{\boldsymbol{i}\boldsymbol{+1}}\boldsymbol{=}\frac{\boldsymbol{1}}{\sqrt{\boldsymbol{d}_{\boldsymbol{i}}\boldsymbol{+1}}}\boldsymbol{h}_{\boldsymbol{i}}\boldsymbol{+}\sum_{\boldsymbol{j=1}}^{\boldsymbol{N}} \frac{\boldsymbol{a}_{\boldsymbol{ij}}}{\sqrt[\boldsymbol{4}]{\left( \boldsymbol{d}_{\boldsymbol{i}}\boldsymbol{+1} \right)\left( \boldsymbol{d}_{\boldsymbol{j}}\boldsymbol{+1} \right)}}\boldsymbol{h}_{\boldsymbol{j}}$. (5)

The VPA mechanism serves to maintain the variance of node embeddings, thus averting feature degradation and alleviating the over-smoothing problem. Concurrently, the VPA mechanism guarantees the stability of embedding variance throughout the information propagation process, thereby addressing the issues of gradient explosion or vanishing that GNNs may encounter during deep propagation.

**Supplementary Section S3: Mathematical Details of the Global Attention Mechanism**

In the bipartite graph ***G*=(*N*,*E*,*X*)**, the initial feature vector $x_{i}$ extracted by ESM2.0, is updated through the following attention mechanism:

$\boldsymbol{Attn}\left( \boldsymbol{H} \right)\boldsymbol{=Softmax}\left( \frac{\boldsymbol{Q}\boldsymbol{K}^{\boldsymbol{T}}}{\sqrt{\boldsymbol{h}}} \right)\boldsymbol{N,Q=H}\boldsymbol{W}_{\boldsymbol{Q}}\boldsymbol{,K=H}\boldsymbol{W}_{\boldsymbol{K}}\boldsymbol{,N=H}\boldsymbol{W}_{\boldsymbol{N}}$, (6)

where $\boldsymbol{H\in}\boldsymbol{R}^{\boldsymbol{m}\boldsymbol{\times f}}$ represents the hidden representation matrix, ***h*** denotes the dimension of the hidden representation, and $\boldsymbol{W}_{\boldsymbol{Q}}\boldsymbol{W}_{\boldsymbol{K}}\boldsymbol{W}_{\boldsymbol{N}}\boldsymbol{\in}\boldsymbol{R}^{\boldsymbol{m}\boldsymbol{\times f}}$ are the trainable weight matrices of the linear projection layer. The attention score matrix $\boldsymbol{A'=Softmax}\left( \frac{\boldsymbol{Q}\boldsymbol{K}^{\boldsymbol{T}}}{\sqrt{\boldsymbol{h}}} \right)$ records the attention weights between all node pairs. Where ***α*** represents the attention score between nodes ***u*** and ***v***. Finally, by multiplying ***A’*** with the node representation matrix ***N***, the model globally updates node features, effectively capturing contextual semantic information and global dependencies.

In TEB prediction, modelling the complex biological relationships between heterogeneous sequences is challenging using only local topological information. To address this, we introduce a global attention mechanism that adaptively integrates global correlation information across different nodes. This mechanism enhances the model's ability to capture cross-scale dependencies, providing a more comprehensive representation of the interactions within the TCR-epitope graph.

**Supplementary Section S4: Formulation of the Deep AUC Maximization Loss**

Given the input space$\boldsymbol{X\in R}$ and output space$\boldsymbol{Y=}\left\{ \boldsymbol{-1,1} \right\}$, the training data originates from an unknown distribution *P*, and following the independent and identical distribution ***Z=X×Y***. The ROC curve is constructed based on the relationship between the True Positive Rate (TPR) and the False Positive Rate (FPR). For any scoring function $\boldsymbol{f}$**:*X→***$\mathbb{R}$, the AUC represents the probability that a positive sample is ranked higher than a negative sample. It is defined as:

$\boldsymbol{AUC}\left( \boldsymbol{f} \right)\boldsymbol{=Pr}\left( \boldsymbol{f}\left( \boldsymbol{x} \right)\boldsymbol{\geq}\boldsymbol{f}\left( \boldsymbol{x'} \right) | \boldsymbol{y=1,y=-1} \right)$, (7)

where $\boldsymbol{f}\left( \boldsymbol{x} \right)$ and $\boldsymbol{f}\left( \boldsymbol{x'} \right)$ denote the scores assigned by the decision function, where *x* represents a positive sample and $\boldsymbol{x'}$ a negative sample, $\boldsymbol{y=1}$and $\boldsymbol{y'=-1}$ represent their corresponding labels. The optimization problem can be reformulated as a stochastic saddle point problem (Nemirovski, et al., 2009). Let $\boldsymbol{m\in}\mathbb{R}^{\boldsymbol{d}}\boldsymbol{,d,e,}\boldsymbol{\tau}\mathbb{\in R,}\boldsymbol{z=}\left( \boldsymbol{x,y} \right)\boldsymbol{\in Z}$, $\boldsymbol{d}$ and $\boldsymbol{e}$ rdenote the average predicted scores for positive and negative samples, respectively, while $\boldsymbol{\tau}$ serves as the hyperparameter that balances the trade-off. Let $\boldsymbol{p}$ denote $\boldsymbol{Pr}\left( \boldsymbol{y=1} \right)$, the probability of a sample belonging to the positive class, and define the Function $\boldsymbol{F}$: ***R^d^***×***R^3^***×***Z***→$\mathbb{R}$ as follows:

$\boldsymbol{F}\left( \boldsymbol{m,d,e,\alpha;z} \right)\boldsymbol{=}\left( \boldsymbol{1-p} \right)\left( \boldsymbol{m}^{\boldsymbol{T}}\boldsymbol{x-d} \right)^{\boldsymbol{2}}\boldsymbol{I}_{\left[ \boldsymbol{y=1} \right]}\boldsymbol{+p}\left( \boldsymbol{m}^{\boldsymbol{T}}\boldsymbol{x-e} \right)^{\boldsymbol{2}}\boldsymbol{I}_{\left[ \boldsymbol{y=-1} \right]}\boldsymbol{+ 2}\left( \boldsymbol{1+\alpha} \right)\left( \boldsymbol{pm}^{\boldsymbol{T}}\boldsymbol{x}\boldsymbol{I}_{\left[ \boldsymbol{y=-1} \right]}\boldsymbol{-}\left( \boldsymbol{1-p} \right)\boldsymbol{m}^{\boldsymbol{T}}\boldsymbol{x}\boldsymbol{I}_{\left[ \boldsymbol{y=1} \right]} \right)\boldsymbol{-p}\left( \boldsymbol{1-p} \right)\boldsymbol{\alpha}^{\boldsymbol{2}}$. (8)

**Supplementary Section S5: Parameter Sensitivity Analysis**

This study performed a parameter sensitivity analysis on the four datasets to assess the stability of the DynaTCR model under varying hyperparameter configurations. The experiments utilized 5-fold cross-validation, with four folds designated as the training set and one fold as the test set. The model incorporates two primary parameters: $\boldsymbol{\gamma}$, which governs the weight of the AUC loss (with 1−$\boldsymbol{\gamma}$ representing the BCE loss weight), and $\boldsymbol{\varepsilon}$, which controls the sampling probability for dynamic edge updates. Parameter ranges were set as follows: $\boldsymbol{\gamma}\boldsymbol{\in}$ {0, 0.1, 0.2, 0.3, 0.4, 0.5, 0.6, 0.7, 0.8, 0.9} and $\boldsymbol{\varepsilon\in}${0.01, 0.02, 0.1, 0.2, 0.3}. With all other hyperparameters and input data held constant, 50 parameter combinations were tested, and results are visualized in **Supplementary Figures s1(a)-(d)**.

Analysis of $\boldsymbol{\gamma}$ reveals that model performance remained relatively stable when $\boldsymbol{\varepsilon}$ ranged from 0 to 0.4, with only minor fluctuations in AUC values, indicating robust tolerance to adjustments in the AUC loss weight. Notably, when $\boldsymbol{\gamma}$ increased to 1 (i.e., the complete exclusion of BCE loss), the AUC score dropped significantly, highlighting the critical role of BCE loss in balancing the joint loss function and enhancing training stability. For dynamic edge sampling probability $\boldsymbol{\varepsilon}$, performance remained stable with slight variations across the tested range (0.01–0.3), demonstrating the DynaTCR model’s strong adaptability to dynamic edge update strategies. These findings collectively indicate that the model exhibits high tolerance to key parameter selection, providing flexibility for hyperparameter tuning.

**Figure s1**. Results of parameter experiments. (a) AUC and (b) AUPR scores of DynaTCR with different $\boldsymbol{\varepsilon}$ values (%). (c) AUC and (d) AUPR scores of DynaTCR with different $\boldsymbol{\gamma}$ values (%).

**Supplementary Section S6: Pre-trained Model Analysis**

This study employs the ESM protein language model to extract initial representations of TCRs and epitopes, facilitating the construction of a TCR-epitope bipartite graph. To assess the impact of different pre-trained models on overall performance, a comparative analysis was conducted using the Transformer-based ESM2.0 and the RNN- and autoencoder-based TCRpeg (Jiang and Li, 2023). ESM2.0, a state-of-the-art biological language model for protein sequence analysis and functional prediction, is pre-trained on large-scale biological sequence data, effectively capturing complex features and functional correlations within protein sequences. In contrast, TCRpeg uses GRU layers to model TCR-epitope relationships and is trained on a specialized dataset containing 106 TCR sequences and 362,456 unique epitopes. However, its generalization ability is constrained by the dataset's limited size.

**Supplementary Figure s2** presents a comparative evaluation of three pre-trained models—ESM2.0 (650M and 8M variants) and TCRpeg under standardized experimental conditions. The results reveal two key findings: First, ESM2-8M outperforms the larger ESM2-650M and the domain-specific TCRpeg. Second, increasing model parameters to 650M does not enhance prediction accuracy, as ESM2-650M performs comparably to TCRpeg. Analysis indicates that the relatively simple interaction patterns of TCR-epitope short sequences make 8 million parameters sufficient for capturing discriminative features, while larger models may overfit task-irrelevant sequence details, impairing generalization.

These findings highlight two important insights. First, protein language models significantly enhance the representational quality of TCR-epitope pairs, underscoring their utility in biological sequence modelling. Second, model size should align with task complexity; the moderate scale of ESM2-8M offers the best cost-effectiveness for TEB prediction.

**Figure s2**. Performance for TCR-epitope graph construction with different pre-trained models.

**Figure s3.** Results of case studies. (a) Comparative contact scores between motif sites and non-motif regions (mean ± 95% confidence interval). The x-axis denotes the motif classification, while the y-axis quantifies the contact score. (b) Performance evaluation of models on an independent dataset. (c–h) predicted contact scores for six CDR3β sequences and their constituent residues within the RSAY motif-containing dataset.

**Supplementary Section 7: Detailed Analysis of Key Contact Sites Detection**

When studying a large number of TCRs targeting a specific epitope, the DynaTCR can effectively predict the binding conformation of TCR with the epitope, thereby revealing the key contact points between TCR and the epitope. This ability is of vital importance for elucidating the mechanism of TCR-epitope interaction. Glanville et al. proposed a strategy to identify enriched CDR3β\betaβ motifs from the TCR library using the GLIPH method.

As shown in Supplementary Figure S3(a), the predicted contact scores of the motif regions identified by GLIPH are significantly higher than those of the non-motif regions, indicating that the DynaTCR model can accurately capture the key contact sites related to epitope binding. Furthermore, in Supplementary Figures S3(c)-(h), six representative cases containing the RSAY motif were analyzed. In these specific instances, the corresponding predicted contact scores of these six CDR3β\betaβ sequences were much higher in the motif regions compared to the non-motif regions.

**Table S1.** Comparative analysis of DynaTCR predictions on Retinitis Pigmentosa-associated antigens.

| **CDR3 Sequence** | **RHO** | **PDE6B** |
| --- | --- | --- |
| CASTGTGVFNEQFF | 0.905 | 0.5789 |
| CASSEGGGEQYF | 0.8657 | 0.6473 |
| CASGGNSYEQYF | 0.8611 | 0.6868 |
| CSTLLAGGGDEQYF | 0.852 | 0.7133 |
| CASSYLPGQGDHYSNQPQHF | 0.8479 | 0.72 |
| CASSQGTGGANEQYF | 0.8479 | 0.72 |
| CASSVAHLNTDTQYF | 0.8443 | 0.7261 |
| CSAPPGPLAHTQYF | 0.8443 | 0.7261 |
| CATSALAGQGRDEQFF | 0.8443 | 0.7261 |
| CASSRLAGTDTQYF | 0.8443 | 0.7261 |
| CASSQGQGLAGIGETQYF | 0.8425 | 0.7281 |
| CASSFKGTGPNQPQHF | 0.8425 | 0.7281 |
| CASSLSGDDYNEQFF | 0.8183 | 0.7325 |
| CASSLIAGNSDTQYF | 0.8183 | 0.7325 |
| CASKGLAGAYTDTQYF | 0.8183 | 0.7325 |
| CASTTSRGAISTDTQYF | 0.7838 | 0.7368 |
| CASSLTSGGPVTDTQYF | 0.7838 | 0.7368 |
| CASSLGGAEAFF | 0.7838 | 0.7368 |
| CASSLGGSTEAFF | 0.7461 | 0.7472 |
| CSADPVAGGGYEQYF | 0.6472 | 0.7764 |

**References**

1.	Dens, C., et al., Interpretable deep learning to uncover the molecular binding patterns determining TCR–epitope interaction predictions. ImmunoInformatics, 2023. 11: p. 100027.

2.	Jiang, F., et al., GTE: a graph learning framework for prediction of T-cell receptors and epitopes binding specificity. Briefings in Bioinformatics, 2024. 25(4): p. bbae343.

3.	Jiang, Y. and S.C. Li, Deep autoregressive generative models capture the intrinsics embedded in t-cell receptor repertoires. Briefings in Bioinformatics, 2023. 24(2): p. bbad038.

Cho, Y.-S. Decoupled variational graph autoencoder for link prediction. In, *Proceedings of the ACM Web Conference 2024*. 2024. p. 839-849.

Jiang, Y. and Li, S.C. Deep autoregressive generative models capture the intrinsics embedded in t-cell receptor repertoires. *Briefings in Bioinformatics* 2023;24(2):bbad038.

Nemirovski, A.*, et al.* Robust stochastic approximation approach to stochastic programming. *SIAM Journal on optimization* 2009;19(4):1574-1609.

Schneckenreiter, L.*, et al.* GNN-VPA: a variance-preserving aggregation strategy for graph neural networks. *arXiv preprint arXiv:2403.04747* 2024.

Zhao, L. and Akoglu, L. Pairnorm: Tackling oversmoothing in gnns. *arXiv preprint arXiv:1909.12223* 2019.
